# Association between mid-upper arm circumference and perceived stress in Chinese adults and older adults: a cross-sectional study

**DOI:** 10.3389/fpubh.2025.1677284

**Published:** 2025-11-21

**Authors:** Yun Li, Lu Ren, Shouzhuan Bao, Duohong Yang

**Affiliations:** 1 School of Physical Education and Sports, Central China Normal University, Wuhan, China; 2 Hubei Sports Vocational College, Wuhan, China; 3 Faculty of Artificial Intelligence in Education, Central China Normal University, Wuhan, China

**Keywords:** mid-upper arm circumference, perceived stress, cross-sectional study, adults, older adults

## Abstract

**Aims:**

This study aimed to explore the relationship between mid-upper arm circumference and perceived stress in Chinese adults and older adults.

**Methods:**

The present study employed cross-sectional data from the CHNS collected during the 2015 survey cycle, involving 8,455 adults and older adults respondents. Perceived stress was assessed utilizing the 14-item Perceived Stress Scale (PSS-14), with scores dichotomized based on the median value. Logistic regression models adjusted for demographic, socioeconomic, and lifestyle variables were applied to examine this associations. To further explore MUAC’s potential as an individual-level indicator, receiver operating characteristic (ROC) analyses were performed.

**Results:**

Compared to participants in the lowest mid-upper arm circumference tertile, those classified within the highest tertile exhibited significantly lower odds of reporting high perceived stress (adjusted OR = 0.71, 95% CI: 0.71–0.93, *p* = 0.002). Subgroup analyses revealed that the inverse association between MUAC and perceived stress was more obvious among adult aged 60 years and older, suburban residents, non-smokers, participants with lower educational levels, and those in the western and northeastern regions. Sensitivity analyses also confirmed the robustness of these findings. The RCS analysis revealed a linear inverse association, with perceived stress declining notably when MUAC exceeded 32.23 cm. ROC analysis indicated that threshold values varied slightly across subgroups, ranging from 27.5–32.4 cm in males (AUC: 0.52–0.60) and 26.2–32.2 cm in females (AUC: 0.51–0.54), with higher values in participants with BMI ≥ 30 kg/m^2^ and slightly higher values in younger males. These findings suggest that MUAC has limited potential for identifying elevated perceived stress and should be regarded as exploratory rather than a validation of its screening utility.

**Conclusion:**

This study indicated that individuals with greater arm circumference tended to report lower stress levels in Chinese adults aged 60 years and older, suggesting exploratory evidence of MUAC’s potential. However, its ability to discriminate perceived stress levels was limited, indicating that MUAC alone is not appropriate as an independent screening tool. It may serve as a simple, low-cost, and complementary indicator in population-based or resource-limited settings, pending further validation in longitudinal studies and integration with other anthropometric or psychosocial measures.

## 1 Introduction

Perceived stress is a well-established psychological construct and serves as a key indicator of mental well-being. Globally, the prevalence of moderate-to-high perceived stress has shown an increasing trend, with approximately 29.6% of adults affected (1). However, prevalence varies significantly across populations and health conditions. For instance, rates as high as 26.7% have been reported among general population in Denmark (2). Relatively lower prevalence rates (6.2–7.8%) have been documented in certain general populations, such as among Swedish adults (3). A survey of adults aged 18 to 65 across 15 provinces in China showed that approximately 47.5% of participants reported a high level of perceived stress (4). Growing evidence suggests that elevated perceived stress levels may contribute to cardiovascular disease (5), diabetes (6), depression (7), cognitive decline (8). Given its widespread prevalence and significant health impacts, identifying modifiable factors associated with perceived stress is critical for developing effective prevention and intervention strategies.

Mid-upper arm circumference is an easily obtainable anthropometric indicator characterized by simplicity, low cost, and convenience (9), and has been documented as capable of substituting other anthropometric measures in effectively predicting perceived stress. Previous research has established associations between arm circumference and various physical health conditions, including type 2 diabetes (10), cardiovascular disease (11), increased systemic inflammation (12), and even mortality (13). However, studies examining the relationship between mid-upper arm circumference and psychological indicators, such as perceived stress, remain extremely limited. To date, only one study has investigated this association, and it was conducted among a specific regional cohort of Turkish college students (14). Furthermore, this prior study utilized a cross-sectional design, restricting causal inference. Perceived stress has been shown to reduce physical activity (15), subsequently decreasing muscle mass (16, 17). Despite these limitations, investigating mid-upper arm circumference as a potential stress marker remains valuable, as it provides an accessible anthropometric measurement that could help identify individuals at higher risk of perceived Stress, informing early intervention strategies. Currently, large-scale representative data from China addressing this topic are lacking. Utilizing data from the China Health and Nutrition Survey (CHNS), this study aims to examine the association between mid-upper arm circumference and perceived stress among Chinese adults and older adults.

## 2 Materials and methods

### 2.1 Study population

The CHNS is a comprehensive longitudinal cohort study initiated to investigate how socioeconomic transitions impact health and nutritional outcomes among Chinese residents. Between 1989 and 2015, 10 survey waves were conducted, employing a multistage random-cluster sampling strategy to recruit participants from rural and urban communities across nine representative provinces, capturing extensive geographic coverage of both northern and southern regions in mainland China. Detailed descriptions of the CHNS methodology and survey protocols have been previously published (18). This collaborative research effort involves the University of North Carolina at Chapel Hill and the National Institute of Nutrition and Food Safety, Chinese Center for Disease Control and Prevention. The dataset from the CHNS is publicly available through the project’s official website^1^.

The study utilized CHNS data from the 2015 survey wave, which included the initial collection of perceived stress data. The final analytic sample was obtained following systematic exclusion criteria applied to the original cohort of 20,226 individuals, based on data completeness, quality, and relevance. The study initially included 12,312 participants. Participants were sequentially excluded for the following reasons: missing perceived stress data (*n* = 2,060), missing educational information (*n* = 1,617), missing alcohol consumption data (*n* = 9), missing smoking data (*n* = 12), missing BMI data (*n* = 15), missing sex information (*n* = 1), abnormal mid-upper arm circumference values (*n* = 108), abnormal BMI values (*n* = 33), and age under 18 years (*n* = 2). After these exclusions, the final analytic sample consisted of 8,455 participants (age range: 18–94 years).

### 2.2 Assessment of perceived stress

The evaluation of perceived stress utilized the perceived stress-14 scale, culturally adapted and linguistically verified in Chinese populations to improve cultural applicability and measurement accuracy (19). Participants rated each item on a five-point Likert scale from 1 (“never”) to 5 (“very often”). The scale includes two distinct components: a negative component (items 1, 2, 3, 8, 11, 12, and 14), assessing frequency of distress associated with stress, and a positive component (items 4, 5, 6, 7, 9, 10, and 13), indicating respondents’ perceived coping capabilities (19). Items with positive wording underwent reverse scoring before aggregation, generating total scores between 14 and 70, wherein higher values represent greater perceived stress. The internal consistency of the perceived stress-14 was found to be acceptable (Cronbach’s *α* = 0.824). To facilitate interpretability in logistic regression analyses, perceived stress score was dichotomized at the median (high vs. low perceived stress). This approach aligns with previous studies conducted in China (20, 21).

### 2.3 Assessment of mid-upper arm circumference

The midpoint measurement of participants’ upper arms was taken midway between the scapular acromion and ulnar olecranon processes, with the individual’s right arm positioned at a 90-degree flexion angle. After fully extending the elbow, mid-upper arm circumference was assessed at this identified midpoint using a flexible metric tape, with measurements recorded to the nearest 0.1 cm. Participants were categorized into tertiles based on mid-upper arm circumference distribution: Tertile 1 (lowest mid-upper arm circumference), Tertile 2 (middle mid-upper arm circumference), and Tertile 3 (highest mid-upper arm circumference).

### 2.4 Covariates

Weight measurements were recorded with participants dressed lightly using a calibrated balance accurate to 0.1 kg. Height was measured to the nearest 0.1 cm using a portable stadiometer, with participants barefoot. Body mass index (BMI) calculation involved dividing weight in kilograms by the square of height in meters (kg/m^2^) (22). BMI was categorized into two groups (≥30 kg/m^2^ vs. <30 kg/m^2^) according to World Health Organization criteria (23). Qualified researchers gathered sociodemographic and lifestyle information through standardized questionnaires covering age (≥60 vs. <60 years) (24), sex, education level (primary, junior high, senior high, vocational, college, postgraduate or higher), residential setting (urban, suburban, county, rural), geographic region (Eastern, Central, Western, Northeastern China), smoking history (smoker or never smoker), and alcohol use (former drinker or non-drinker).

### 2.5 Statistical methods

Baseline participant demographics were summarized by tertiles of mid-upper arm circumference using descriptive statistical methods. Continuous variables were presented as means and 95% confidence intervals (CIs), with intergroup differences assessed by ANOVA. Categorical variables were summarized using frequencies and percentages, with chi-square (*χ*^2^) tests evaluating differences between groups.

Mid-upper arm circumference tertiles and perceived stress were used as the independent and dependent variables. Generalized linear models (GLMs) were employed to investigate the relationship between mid-upper arm circumference and perceived stress (continuous variables). Logistic regression analyses explored the binary classification of perceived stress (high versus low), calculating odds ratios (ORs) and their corresponding 95% CIs through maximum likelihood methods. Models included adjustments for potential confounders such as age, sex, BMI, smoking and drinking, education level, residential region, and geographic region. Subgroup analyses stratified by these covariates were conducted to examine potential modifications of effects. To evaluate the utility of mid-upper arm circumference in classifying perceived stress status, we divided the cohort into high- and low-stress groups using a median split and performed a ROC analysis. The analysis by gender and age (≥60 vs. <60 years) using the United Nations definition of older adults (24) and further conducted according to BMI categories (≥30 kg/mm^2^ vs. <30 kg/m^2^). Predictive accuracy was evaluated using the AUC, where a higher AUC value indicates better predictive performance.

Restricted cubic spline (RCS) models were utilized via R software to assess possible non-linear associations between mid-upper arm circumference and perceived stress. The spline analyses incorporated three knots placed at the 10th, 50th, and 90th percentiles of mid-upper arm circumference distribution, facilitating flexible characterization of dose–response relationships. Stata version 16.0 (Stata Corp LLC) was employed for all other statistical evaluations, with statistical significance determined at a two-tailed *p*-value below 0.05.

## 3 Results

Table 1 summarizes participant characteristics stratified by mid-upper arm circumference tertiles. A clear gradient was observed for several variables. Participants in higher mid-upper arm circumference tertiles were older and more likely to be male. The prevalence of both smoking and alcohol consumption also increased progressively across mid-upper arm circumference tertiles. Similarly, BMI was systematically higher with increasing mid-upper arm circumference. Residential patterns varied, with a greater proportion of urban residents in the highest tertile. Significant variations were also noted in educational attainment and geographic distribution, where higher mid-upper arm circumference was associated with a higher representation of participants from eastern regions and those with higher educational attainment.

**Table 1:** Participant characteristics by mid-upper arm circumference category ^a^.

Variable	Tertile 1	Tertile 2	Tertile 3	χ/*F*	*p* *^a^*
Age range, years	18–94	18–92	18–94	—	—
Age, years	49.8 (49.2–50.4)	51.5 (51.0–52.0)	51.3 (50.8–51.8)	12.86	<0.001
Sex (male), %	42.0	49.6	55.6	103.0	<0.001
Smoker, %	24.2	27.7	28.7	15.9	<0.001
Drinker, %	23.8	30.2	32.8	57.8	<0.001
Residential regions, %					
Urban	23.7	24.7	30.8	53.3	<0.001
Suburban	15.3	14.8	14.8		
County	17.8	20.3	18.1		
Rural	43.2	40.2	36.3		
BMI, kg/m^2^	21.7 (21.6–21.8)	24.3 (24.2–24.4)	26.9 (26.8–27.0)	2357.11	<0.001
Educational level, %					
Primary school	20.1	21.8	16.7	28.0	0.002
Junior high school	37.6	38.1	39.1		
Senior high school	16.5	16.6	17.8		
Vocational school	9.6	8.9	10.1		
College	15.7	14.0	15.7		
Master’s degree or above	0.5	0.6	0.7		
Geographic region, %					
Eastern	27.6	35.6	42.4	352.7	<0.001
Central	26.1	24.3	21.8		
Western	33.4	22.6	14.9		
Northeastern	12.9	17.5	20.9		

a Comparisons between groups were performed using one-way ANOVA for continuous variables and chi-squared tests for categorical variables.

Tertiles (1-3) for mid-upper arm circumference were created by dividing the study population into three equal groups based on the MUAC value distribution. Specifically, Tertile 1 represents the group with the lowest MUAC values, Tertile 2 the middle group, and Tertile 3 the group with the highest values. This grouping method is applied consistently in the tables that follow.

The results of the logistic regression analyses are presented in Table 2. In the crude model (Model 1), a significant inverse association between mid-upper arm circumference tertiles and perceived stress was observed. Compared with the reference group (Tertile 1), the unadjusted odds ratios (ORs) were 0.90 (95% CI: 0.81, 1.00) for Tertile 2 and 0.81 (95% CI: 0.71, 0.87) for Tertile 3, indicating a significant inverse trend across tertiles (*p* for trend < 0.001). After adjusting for multiple potential confounders including age, gender, BMI, smoking status, alcohol consumption, stratum, educational attainment, economic level, and community category (Model 2), the inverse association was attenuated but remained statistically significant for the highest tertile. Specifically, the adjusted OR for Tertile 2 was 0.90 (95% CI: 0.81, 1.01), which was not significant. However, participants in the highest mid-upper arm circumference tertile (Tertile 3) showed significantly reduced odds of experiencing high perceived stress (adjusted OR = 0.71; 95% CI: 0.71, 0.93). The *p*-value for the linear trend across tertiles in the fully adjusted model was 0.002, indicating a persistently significant inverse dose–response relationship between mid-upper arm circumference and the risk of perceived stress after multivariable adjustments.

**Table 2:** Logistic regression analysis of the association between mid-upper arm circumference and risk of perceived stress.

*N* = 8,455	Mean value of mid-upper arm circumference	Number of perceived stress	Model 1^**a**^	Model 2^**b**^
Mid-upper arm circumference	27.9 ± 3.7	—	—	—
Tertile 1 (*n* = 2,783)	24.0 ± 2.2	1,545	1.000 (reference)	1.000 (reference)
Tertile 2 (*n* = 2,915)	27.8 ± 0.8	1,540	0.90 (0.81, 1.00)^c^	0.90 (0.81, 1.01)
Tertile 3 (*n* = 2,757)	31.8 ± 2.3	1,365	0.81 (0.71, 0.87)	0.71 (0.71, 0.93)
*p* for trend^d^	—	—	<0.001	0.002

a Model 1: unadjusted.

b Model 2: further adjusted for age, sex, BMI, smoking and drinking, education level, residential region, and geographic region.

c Adjusted data are expressed as OR (95% CI).

d *p* for trend were obtained using multivariate logistic regression analyses.

To further explore population heterogeneity, subgroup analyses were conducted to examine whether the association between MUAC and perceived stress differed across demographic and lifestyle characteristics. As shown in Table 3, the association was stronger in adult aged 60 years and older and those residing in suburban areas. Significant inverse trends were also observed among non-smokers, implying that adverse lifestyle factors may attenuate the protective relationship between MUAC and perceived stress. Further analysis identified regional variations, with significantly stronger inverse associations observed in the western and northeastern regions. The relationship was also more evident in participants with lower educational levels. Taken together, these findings demonstrate that multiple sociodemographic and lifestyle factors, in addition to sex, BMI and drinking status, modify the association between MUAC and perceived stress, underscoring the presence of effect modification across different population subgroups.

**Table 3:** Subgroup analyses of the association between mid-upper arm circumference and risk of perceived stress.

Subgroups	Tertiles of MUAC	Model 1^a^	Model 2^b^
Sex			
Men (*n* = 4,148)	Tertile 1 (*n* = 1,625)	Reference	Reference
	Tertile 2 (*n* = 1,435)	0.90 (0.78, 1.04) ^c^	0.93 (0.80, 1.09)
	Tertile 3 (*n* = 1,088)	0.75 (0.64, 0.87)	0.80 (0.66, 0.97)
	*p* for trend^c^	<0.001	0.023
Women (*n* = 4,307)	Tertile 1 (*n* = 1,614)	Reference	Reference
	Tertile 2 (*n* = 1,469)	0.93 (0.81, 1.07)	0.91 (0.78, 1.06)
	Tertile 3 (*n* = 1,224)	0.83 (0.72, 0.97)	0.82 (0.68, 0.99)
	*p* for trend^c^	0.017	0.041
Age, years			
18–40 (*n* = 2022)	Tertile 1 (*n* = 757)	Reference	Reference
	Tertile 2 (*n* = 605)	1.03 (0.83, 1.27)	0.98 (0.78, 1.23)
	Tertile 3 (*n* = 660)	1.01 (0.81, 1.26)	0.94 (0.69, 1.27)
	*p* for trend^c^	0.944	0.678
41-60 (*n* = 4,174)	Tertile 1 (*n* = 1,661)	Reference	Reference
	Tertile 2 (*n* = 1,150)	0.95 (0.81, 1.10)	0.98 (0.84, 1.15)
	Tertile 3 (*n* = 1,363)	0.84 (0.73, 0.97)	0.95 (0.79, 1.13)
	*p* for trend^c^	0.020	0.552
≥60 (*n* = 2,503)	Tertile 1 (*n* = 837)	Reference	Reference
	Tertile 2 (*n* = 877)	0.96 (0.79, 1.16)	1.02 (0.83, 1.25)
	Tertile 3 (*n* = 789)	0.66 (0.54, 0.80)	0.73 (0.57, 0.93)
	*p* for trend^c^	<0.001	0.009
BMI, kg/m^2^			
BMI > = 30 (*n* = 508)	Tertile 1 (*n* = 190)	Reference	Reference
	Tertile 2 (*n* = 149)	0.68 (0.44, 1.04)	0.62 (0.40, 0.97)
	Tertile 3 (*n* = 169)	0.73 (0.48, 1.10)	0.58 (0.37, 0.92)
	*p* for trend^c^	0.122	0.017
BMI < 30 (*n* = 7,947)	Tertile 1 (*n* = 2,768)	Reference	Reference
	Tertile 2 (*n* = 2,865)	0.89 (0.80, 0.99)	0.89 (0.79, 1.00)
	Tertile 3 (*n* = 2,314)	0.79 (0.71, 0.88)	0.81 (0.71, 0.93)
	*p* for trend^c^	<0.001	0.003
Smoking status			
Non-smokers (*n* = 6,181)	Tertile 1 (*n* = 2,109)	Reference	Reference
	Tertile 2 (*n* = 2,107)	0.93 (0.83, 1.05)	0.90 (0.79, 1.03)
	Tertile 3 (*n* = 1965)	0.78 (0.69, 0.89)	0.76 (0.65, 0.89)
	*p* for trend^c^	<0.001	0.001
Smokers (*n* = 2,274)	Tertile 1 (*n* = 944)	Reference	Reference
	Tertile 2 (*n* = 574)	0.84 (0.68, 1.04)	0.89 (0.71, 1.12)
	Tertile 3 (*n* = 756)	0.83 (0.68, 1.00)	0.92 (0.72, 1.18)
	*p* for trend^c^	0.050	0.515
Drinking status			
Non-drinkers (*n* = 6,008)	Tertile 1 (*n* = 2,120)	Reference	Reference
	Tertile 2 (*n* = 2036)	0.92 (0.81, 1.04)	0.90 (0.79, 1.03)
	Tertile 3 (*n* = 1852)	0.80 (0.70, 0.90)	0.80 (0.68, 0.94)
	*p* for trend^c^	<0.001	0.006
Drinkers (*n* = 2,447)	Tertile 1 (*n* = 951)	Reference	Reference
	Tertile 2 (*n* = 849)	0.86 (0.71, 1.04)	0.87 (0.71, 1.07)
	Tertile 3 (*n* = 647)	0.76 (0.62, 0.93)	0.77 (0.60, 0.99)
	*p* for trend^c^	0.007	0.044
Residential area			
Urban (*n* = 2,228)	Tertile 1 (*n* = 746)	Reference	Reference
	Tertile 2 (*n* = 870)	1.01 (0.83, 1.23)	1.04 (0.84, 1.28)
	Tertile 3 (*n* = 612)	0.89 (0.72, 1.10)	0.92 (0.71, 1.19)
	*p* for trend^c^	0.310	0.563
Suburban (*n* = 1,265)	Tertile 1 (*n* = 425)	Reference	Reference
	Tertile 2 (*n* = 431)	0.77 (0.59, 1.01)	0.73 (0.54, 0.98)
	Tertile 3 (*n* = 409)	0.60 (0.46, 0.79)	0.52 (0.37, 0.74)
	*p* for trend^c^	<0.001	<0.001
County (*n* = 1,585)	Tertile 1 (*n* = 531)	Reference	Reference
	Tertile 2 (*n* = 556)	0.99 (0.77, 1.25)	0.94 (0.72, 1.22)
	Tertile 3 (*n* = 498)	0.99 (0.78, 1.27)	0.91 (0.66, 1.25)
	*p* for trend^c^	0.958	0.550
Rural (*n* = 3,377)	Tertile 1 (*n* = 1,203)	Reference	Reference
	Tertile 2 (*n* = 1,172)	0.88 (0.75, 1.04)	0.90 (0.75, 1.08)
	Tertile 3 (*n* = 1,002)	0.78 (0.66, 0.92)	0.84 (0.67, 1.05)
	*p* for trend^c^	0.004	0.120
Geographical region			
Eastern (*n* = 2,976)	Tertile 1 (*n* = 1,091)	Reference	Reference
	Tertile 2 (*n* = 1,046)	1.05 (0.89, 1.24)	1.03 (0.86, 1.24)
	Tertile 3 (*n* = 839)	1.05 (0.87, 1.25)	1.04 (0.83, 1.30)
	*p* for trend^c^	0.602	0.751
Central (*n* = 2036)	Tertile 1 (*n* = 727)	Reference	Reference
	Tertile 2 (*n* = 708)	0.86 (0.69, 1.06)	0.87 (0.69, 1.09)
	Tertile 3 (*n* = 601)	0.84 (0.67, 1.05)	0.85 (0.65, 1.12)
	*p* for trend^c^	0.112	0.245
Western (*n* = 1998)	Tertile 1 (*n* = 713)	Reference	Reference
	Tertile 2 (*n* = 689)	0.93 (0.75, 1.15)	1.03 (0.81, 1.31)
	Tertile 3 (*n* = 596)	1.10 (0.88, 1.38)	1.38 (1.02, 1.88)
	*p* for trend^c^	0.437	0.041
Northeastern (*n* = 1,445)	Tertile 1 (*n* = 521)	Reference	Reference
	Tertile 2 (*n* = 538)	0.93 (0.73, 1.19)	0.80 (0.61, 1.04)
	Tertile 3 (*n* = 386)	0.70 (0.54, 0.92)	0.53 (0.37, 0.75)
	*p* for trend^c^	0.013	<0.001
Education level			
Primary school (*n* = 1,655)	Tertile 1 (*n* = 559)	Reference	Reference
	Tertile 2 (*n* = 635)	0.89 (0.71, 1.12)	0.90 (0.70, 1.16)
	Tertile 3 (*n* = 461)	0.74 (0.58, 0.95)	0.77 (0.56, 1.06)
	*p* for trend^c^	0.020	0.108
Junior high school (*n* = 3,234)	Tertile 1 (*n* = 1,129)	Reference	Reference
	Tertile 2 (*n* = 1,028)	0.83 (0.70, 0.99)	0.86 (0.72, 1.03)
	Tertile 3 (*n* = 1,077)	0.72 (0.61, 0.85)	0.77 (0.62, 0.95)
	*p* for trend^c^	<0.001	0.017
Senior high school (*n* = 1,435)	Tertile 1 (*n* = 491)	Reference	Reference
	Tertile 2 (*n* = 468)	0.94 (0.73, 1.21)	0.93 (0.71, 1.22)
	Tertile 3 (*n* = 476)	0.72 (0.56, 0.93)	0.75 (0.55, 1.02)
	*p* for trend^c^	0.011	0.072
Vocational school (*n* = 803)	Tertile 1 (*n* = 268)	Reference	Reference
	Tertile 2 (*n* = 270)	0.90 (0.64, 1.26)	0.83 (0.57, 1.21)
	Tertile 3 (*n* = 265)	0.85 (0.61, 1.20)	0.69 (0.44, 1.08)
	*p* for trend^c^	0.358	0.103
College (*n* = 1,278)	Tertile 1 (*n* = 437)	Reference	Reference
	Tertile 2 (*n* = 421)	1.06 (0.81, 1.39)	1.02 (0.76, 1.36)
	Tertile 3 (*n* = 420)	1.18 (0.91, 1.55)	1.04 (0.74, 1.47)
	*p* for trend^c^	0.217	0.818
Master’s degree or above (*n* = 50)	Tertile 1 (*n* = 19)	Reference	Reference
	Tertile 2 (*n* = 17)	1.52 (0.39, 5.95)	3.28 (0.63, 17.2)
	Tertile 3 (*n* = 14)	1.63 (0.39, 6.82)	3.74 (0.50, 28.07)
	*p* for trend^c^	0.493	0.161

a Model 1: unadjusted.

b Model 2: further adjusted for age, sex, BMI, smoking and drinking, education level, residential region, and geographic region.

c Adjusted data are expressed as OR (95% CI).

Sensitivity analyses remained robust when a general linear model was employed, treating perceived stress as a continuous variable (Table 4).

**Table 4:** Generalized linear model analysis of the association between mid-upper arm circumference and perceived stress score.

*N* = 8,455	*β* coefficients (95% CI)	*Z* value	*p*-value
Model 1^a^			
Tertile 1 (*n* = 2,783)	Reference		
Tertile 2 (*n* = 2,915)	−0.331 (−0.648, −0.013)^c^	−2.04	0.041
Tertile 3 (*n* = 2,757)	−0.766 (−1.093, −0.439)	−4.59	0.000
Model 2^b^			
Tertile 1 (*n* = 2,783)	Reference		
Tertile 2 (*n* = 2,915)	−0.248 (−0.587, 0.009)	−1.44	0.151
Tertile 3 (*n* = 2,757)	−0.544 (−0.952, −0.135)	−2.61	0.009

a Model 1: unadjusted.

b Model 2: further adjusted for age, sex, BMI, smoking and drinking, education level, residential region, and geographic region.

c Adjusted data are expressed as β coefficients (95%CI).

The ROC analysis indicated the cut-off values varied slightly across subgroups, ranging from 27.5 cm to 32.4 cm in males (AUC: 0.52–0.60) and from 26.2 cm to 32.2 cm in females (AUC: 0.51–0.54). Overall, cut-off values were higher in participants with a BMI ≥ 30 kg/m^2^ compared to those with a BMI < 30 kg/mm^2^ across all sex and age categories, with a tendency for younger males to have slightly higher values. These found indicated that MUAC may have limited potential for identifying individuals with elevated perceived stress. These findings should be interpreted as exploratory evidence rather than a validation of MUAC’s screening utility (data not shown).

**Figure 1:**
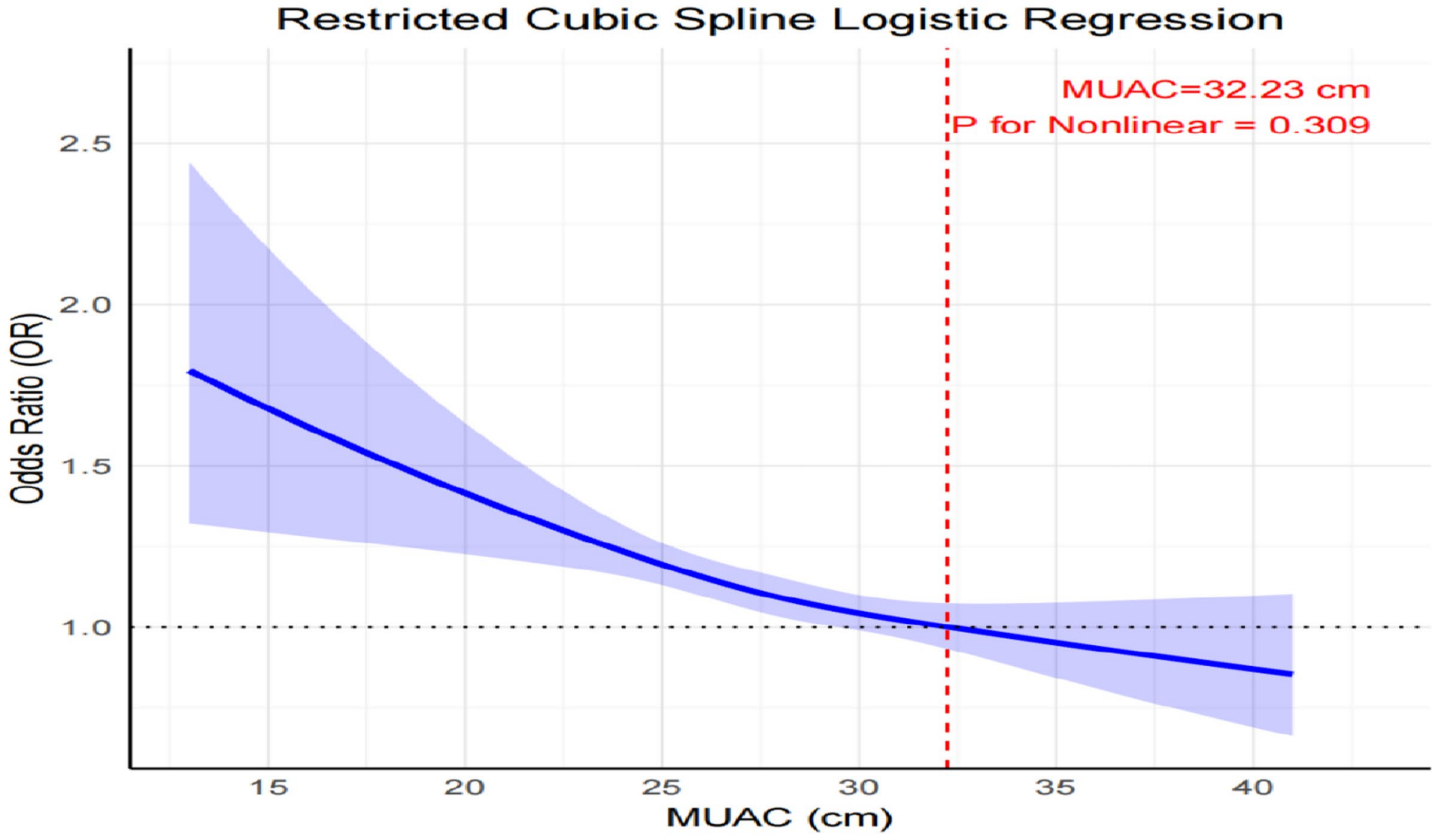
Cubic model of the association between mid-upper arm circumference and risk of perceived stress after adjusting for sex, age, BMI, smoking, drinking, residential area, geographical region, and education level. The graph plots the risk of perceived stress (on the y-axis) against the measured mid-upper arm circumference, in centimeters (on the x-axis).

The RCS analysis was utilized to flexibly model the dose–response relationship between mid-upper arm circumference and perceived stress. The analysis did not reveal significant nonlinearity (*p* for nonlinearity = 0.309), suggesting a linear association between mid-upper arm circumference and perceived stress across the observed mid-upper arm circumference range. Specifically, increasing mid-upper arm circumference values corresponded to progressively lower perceived stress levels, with 32.23 specific reference values (Figure 1).

## 4 Discussion

This cross-sectional analysis of Chinese adults and older adults examine the relationship between MUAC and perceived stress. We observed an inverse association between MUAC and perceived stress in multivariable analyses. Moreover, the inverse association between MUAC and perceived stress was more pronounced among adult aged 60 years and older, suburban residents, non-smokers, participants with lower educational levels, and those in the western and northeastern regions. To our knowledge, this study provides novel large-scale evidence on the association between mid-upper arm circumference and perceived stress in Chinese adults and older adults, highlighting mid-upper arm circumference as an accessible anthropometric biomarker linking musculoskeletal health with psychosocial well-being.

Several studies conducted among younger adults reported a positive correlation between mid-upper arm circumference and perceived stress (14). However, these results contrast with the findings of the present study. One plausible explanation for this discrepancy is the differing composition of mid-upper arm circumference across age groups, as younger adults typically have a higher proportion of fat mass (14, 25), while muscle mass constitutes a larger proportion of arm circumference in middle-aged adults (26).

This study explored two plausible biological mechanisms underlying the observed association between mid-upper arm circumference and perceived stress. Firstly, individuals with higher mid-upper arm circumference had significantly lower perceived stress, suggesting a potential role of skeletal muscle in stress regulation. Skeletal muscle functions as an active endocrine organ, influencing neurobiological processes through secretion of myokines and modulation of neurotransmitter activity. For instance, physical activity and muscle metabolism enhance the synthesis and release of neuromodulators such as serotonin (5-HT) and dopamine (DA), both of which contribute to emotional regulation and stress resilience (27, 28). From a neurobiological perspective, elevated muscle mass may aid in stress regulation through modulating neurotransmitter systems that inhibit hypothalamic–pituitary–adrenal (HPA) axis overactivation and cortisol secretion, consequently lowering perceived stress (29). Additionally, skeletal muscles release bioactive factors such as myokines, notably brain-derived neurotrophic factor (BDNF), which enhance neural plasticity, support hippocampal neurogenesis, and confer neuroprotection (30). BDNF further moderates stress responses by attenuating HPA-axis reactivity, promoting emotional resilience, and alleviating psychological distress (31).

This study has several inherent limitations. Firstly, the cross-sectional study design restricts causal interpretations of the observed associations, making the temporal relationship between mid-upper arm circumference and perceived stress uncertain. Exercise level or engagement in sports activities represents another critical confounder, as it can influence both muscle mass (32, 33) and mental health outcomes (34, 35). Prospective longitudinal and intervention studies are warranted to elucidate these causal pathways further. Secondly, although the sample was derived from a nationally representative Chinese population, the findings’ applicability to other demographic contexts remains uncertain, necessitating validation through additional international research. Thirdly, mid-upper arm circumference acted as an indirect indicator of muscle mass; however, direct measurements of body composition via dual-energy X-ray absorptiometry (DXA) or bioelectrical impedance analysis (BIA) were unavailable, potentially reducing accuracy in assessing muscular development. This limitation weakens biological interpretation, as mid-upper arm circumference may reflect subcutaneous fat or overall body size in addition to muscle mass (36). Future studies incorporating precise body composition assessments are warranted to validate these associations. Fourth, while the Chinese version of the perceived stress demonstrated satisfactory cultural validity, the inherent subjectivity in self-reported stress measures may introduce potential information bias. Fifth, a substantial number of participants were excluded from the analysis. All demographic and lifestyle variables showed significant differences between the included and excluded samples. These demographic and lifestyle differences between included and excluded participants should be considered when interpreting the generalizability of the findings (Appendix Table 2). Sixth, mid-upper arm circumference was measured only among adults aged 18 and older; thus, the relationship between mid-upper arm circumference and perceived stress in younger populations was not examined. Future research should include younger adults to confirm this association. Seventh, an additional issue to be considered in this study is the potential confounding effect of socioeconomic status (SES) on the association between mid-upper arm circumference and PSS. It is well-established that SES is a recognized determinant of both physical and mental health. Socioeconomic status, typically reflected by income and educational attainment (37), is often associated with increased exposure to chronic stressors among lower income individuals, such as financial insecurity (38), employment instability (39). These stressors may contribute to elevated levels of perceived stress (38, 40). Concurrently, SES may also influence nutritional status. Individuals with lower SES often have limited access to nutrient-rich foods (41, 42), which can lead to undernutrition or reduced muscle mass (43, 44), thereby resulting in a smaller mid-upper arm circumference. Conversely, those with lower SES tend to consume diets high in low-cost, energy-dense foods (45), which may also increase the risk of obesity (46). Thus, SES may exert an adverse influence on mid-upper arm circumference through dietary pathways. Given these complex interrelationships, the observed association between mid-upper arm circumference and perceived stress in this study may partly reflect underlying SES. Although adjustments were made for education and region, residual confounding cannot be entirely ruled out. Future studies incorporating more detailed SES indicators, such as household income and occupation, are warranted to clarify the independent effect of mid-upper arm circumference on perceived stress after accounting for socioeconomic influences. Finally, since perceived stress is self-reported, recall bias cannot be avoided. Future studies are warranted to determine whether mid-upper arm circumference can provide incremental or complementary clinical utility beyond self-reported stress measures and to explore causal relationships between mid-upper arm circumference and perceived stress.

## 5 Clinical implications and limitations of MUAC as a predictor of perceived stress

In this cross-sectional sample of Chinese adults, MUAC exhibited only a weak-to-modest capacity to discriminate levels of perceived stress, with AUC values predominantly ranging between 0.51 and 0.60. Therefore, MUAC should not be regarded as an independent clinical screening tool for psychological stress. Instead, the present findings indicate that MUAC may serve as an exploratory and complementary anthropometric indicator in population-based studies, particularly when simple and rapid field assessments are required, or when combined with other anthropometric or perceived stress.

From a practical perspective, MUAC may offer useful insights at the group or population level or as part of a composite prediction models, but it does not meet the threshold for individual-level clinical decision-making in most subgroups. It may be particularly valuable in large-scale epidemiological studies, resource-limited settings, or longitudinal research exploring anthropometric-psychosocial relationships, provided its limitations are clearly recognized. In the use of MUAC, researchers should not propose a single diagnostic cut-off unless it has been externally validated using an independent cohort or a longitudinal prediction model. This underscores the need for future studies to validate MUAC cut-offs in larger prospective cohorts and to explore whether combining MUAC with other variables, to improve perceived stress screening accuracy.

## 6 Conclusion

This study indicated that individuals with greater arm circumference tended to report lower stress levels in Chinese adults aged 60 years and older, suggesting exploratory evidence of MUAC’s potential. However, its ability to discriminate perceived stress levels was limited, indicating that MUAC alone is not appropriate as an independent screening tool. It may serve as a simple, low-cost, and complementary indicator in population-based or resource-limited settings, pending further validation in longitudinal studies and integration with other anthropometric or psychosocial measures.

## Data Availability

The raw data supporting the conclusions of this article will be made available by the authors, without undue reservation.

## Footnotes

^1^ https://www.cpc.unc.edu/projects/china/data/datasets/longitudinal
