# Supplementary material for: Association between mid-upper arm circumference and perceived stress in Chinese adults and older adults: a cross-sectional study
Source: Front Public Health. 2025 Nov 21;13:1677284. doi: 10.3389/fpubh.2025.1677284 (PMC12678243; doi:10.3389/fpubh.2025.1677284)
Supplement: Supplementary file 1 [file Table_1.DOCX]

**Appendix Table 1 Adjusted Mean Perceived Stress Scores Across MUAC Tertiles Among Adults**

| **N=8455** | **Adjusted mean (95%CI)** | **P for trend** |
| --- | --- | --- |
| **Model 1** **^a^** |  |  |
| Tertile 1 (n = 2783) | 36.85（36.62～37.08） | <0.001 |
| Tertile 2 (n = 2915) | 36.50（36.27～36.72） |  |
| Tertile 3 (n = 2757) | 36.05（35.82～36.28） |  |
| **Model 2** **^b^** |  |  |
| Tertile 1 (n = 2783) | 36.73 (36.47～36.99) | 0.009 |
| Tertile 2 (n = 2915) | 36.48（36.26～36.70） |  |
| Tertile 3 (n = 2757) | 36.19（35.93～36.45） |  |

^a^ Model 1：crude

^b^ Model 2：was additionally adjusted for sex, age, BMI, smoking, drinking, residential area, geographical region, and education level.
