# Supplementary material for: Association between mid-upper arm circumference and perceived stress in Chinese adults and older adults: a cross-sectional study
Source: Front Public Health. 2025 Nov 21;13:1677284. doi: 10.3389/fpubh.2025.1677284 (PMC12678243; doi:10.3389/fpubh.2025.1677284)
Supplement: Supplementary file 2 [file Table_2.DOCX]

**Appendix Table 2. Comparison of Demographic and Lifestyle Characteristics Between Excluded and Included Participants​​ ^a^**

| Variable | **Included** | **Excluded** | **Χ^2^/t** | ***P ^a^*** |
| --- | --- | --- | --- | --- |
| **Age, years** | 50.9 (50.6, 51.2) | 37.7 (37.1, 38.2) | 41.341 | <0.001 |
| **Sex (male), %** | 49.1 | 46.7 | 12.034 | 0.021 |
| **Smoking, %** | 26.9 | 23.3 | 20.521 | <0.001 |
| **Drinking, %** | 28.9 | 23.7 | 40.963 | <0.001 |
| **Residential Region, %** |  |  |  |  |
| Urban | 26.4 | 16.5 | 353.755 | <0.001 |
| Suburban | 15.0 | 16.0 |  |  |
| County | 18.8 | 15.4 |  |  |
| Rural | 39.9 | 52.2 |  |  |
| **BMI, kg/m^2^** | 24.3 (24.2, 24.3) | 22.3 (22.1, 22.4) | 23.043 | <0.001 |
| **Educational Attainment, %** |  |  | 28.004 | <0.001 |
| Primary school | 19.6 | 22.9 |  |  |
| Junior high school | 38.3 | 37.1 |  |  |
| Senior high school | 17.0 | 15.9 |  |  |
| Vocational school | 9.5 | 7.7 |  |  |
| College | 15.1 | 15.7 |  |  |
| Master's degree or above | 0.6 | 0.7 |  |  |
| **Geographic Region, %** |  |  | 842.834 | <0.001 |
| Eastern | 35.2 | 28.5 |  |  |
| Central | 24.1 | 21.3 |  |  |
| Western | 23.6 | 41.6 |  |  |
| Northeastern | 17.1 | 8.6 |  |  |

**^a^ Comparisons between groups were conducted using independent-sample t-tests for continuous variables and chi-squared tests for categorical variables.**
